# Acute Effects of Moderate versus High-Intensity Strength Exercise on Attention and Mood States in Female Physical Education Students

**DOI:** 10.3390/life11090931

**Published:** 2021-09-07

**Authors:** Hela Znazen, Maamer Slimani, Atyh Hadadi, Turki Alzahrani, David Tod, Nicola Luigi Bragazzi, Nizar Souissi

**Affiliations:** 1 Department of Physical Education and Sport, College of Education, Taif University, P.O. Box 11099, Taif 21944, Saudi Arabia; znazen.hela@laposte.net (H.Z.); Atyhhadadi@gmail.com (A.H.); tm.alzahrani@tu.edu.sa (T.A.); 2 Department of Health Sciences (DISSAL), Postgraduate School of Public Health, Genoa University, 16132 Genoa, Italy; 3 Higher Institute of Sport and Physical Education of Ksar Said, University of Manouba, Manouba 2037, Tunisia; 4 School of Sport and Exercise Science, Liverpool John Moores University, Liverpool L3 3AF, UK; D.A.Tod@ljmu.ac.uk; 5 Laboratory for Industrial and Applied Mathematics (LIAM), Department of Mathematics and Statistics, York University, Toronto, ON M3J 1P3, Canada; robertobragazzi@gmail.com; 6 Tunisian Research Laboratory ‘‘Sports Performance Optimization’’, National Center of Medicine and Science in Sports (CNMSS), El Menzah, Tunis 1004, Tunisia; n_souissi@yahoo.fr

**Keywords:** exercise, intensity, attention, RPE, mood

## Abstract

The presumed benefits of exercise/physical activity on the brain are an important public health issue. However, the experimental approach to understanding the effects of physical activity on the brain, and more particularly on cognitive functions, has only been studied recently. In particular, females remain underrepresented in the research, despite having a specific training/exercise adaptation/response. The aim of the present study was to examine the acute effects of high- and moderate-intensity strength exercise (3 sets of 8–10 repetitions and 3 sets of 6 repetitions, respectively, with each session lasting approximately 30 min) on attention and mood states in female physical education students. Forty-six female physical education students (M_age_ = 20.02 ± 1.05 years, M_Body Mass Index_ = 21.07) volunteered to participate in this study. They were divided into three groups: a moderate-intensity strength exercise group (MISEG: *n* = 15), a high-intensity strength exercise group (HISEG: *n* = 16), and a control group (CG: *n* = 15). Attention and psychological states were assessed using the d2 test, Rating of Perceived Exertion (RPE) and the Brunel Mood Scale (BRUMS) questionnaire, respectively, before and after each session. The data showed that in the MISEG attention increased, in terms of concentration (*p* = 0.05). RPE values, fatigue and confusion were higher for the HISEG than the CG (*p* < 0.05) and the MISEG (*p* < 0.05). Vigour was higher for the MISEG than other groups (*p* < 0.05). In conclusion, moderate-intensity resistance exercise is an appropriate method to improve attention in female participants. The elevated cognitive performance may be due to the changes in RPE and mood states (fatigue, vigour and confusion subscales).

## 1. Introduction

The presumed benefits of exercise/physical activity on the brain are an important health issue. However, the experimental approach to understanding the effects of physical activity on the brain, and more particularly on cognitive functions, has only occurred recently. The most obvious benefits are seen for the elderly, where physical activity slows the rate of cognitive decline associated with ageing [1,2,3,4]. The size of the effect of physical activity on cognitive functioning is dependent upon a number of factors, such as the type of cognitive performance assessed, exercise intensity, exercise duration, and participant fitness [5]. More in-depth knowledge about the relationship between exercise/physical activity and cognitive functioning could inform researchers, in particular sports scientists and neuroscientists, as well as physicians.

An increasing number of scholarly studies have been designed to understand the beneficial effects of acute exercise on cognitive function and psychological states. To our knowledge, Gabbard and Barton [6] were the first authors who examined this issue. They found an improvement in the ability to solve mathematical problems in seven and eight-year-old students after 50 min of physical education. However, the same physical education course of 20, 30 or 40 min duration proved to be ineffective. Recently, it has been demonstrated that both moderate- and high-intensity exercise increases cognitive function after acute aerobic, strength and intermittent exercises [7,8,9,10,11]. The optimal effects of aerobic exercise on cognitive function range between 60 and 70% of maximum heart rate, which is defined as moderate-intensity [12,13,14].

The effect of acute exercise on cognitive function has been summarized in many reviews using statistical meta-analysis techniques [15,16,17]. These reviews have reported a small but significant beneficial effect of acute exercise on cognitive function. However, most of the studies have examined the effect of aerobic, intermittent, walking, and yoga exercise on cognitive performance [15,16,17]. Few studies have investigated the effect of different strength exercise intensities on cognitive function [18,19,20]. Further, few of these studies have involved female-only samples (8.3%), whereas the majority (about 25% and 58.3%) have included male and mixed participant samples [21]. A study recruiting women only showed that, after the aerobic and strength intervention, some tasks and executive functions (measured by the Stroop “non-color word” and “color word” test) but not others (assessed by the Trail Making Test) were improved [7]. Further, the previous authors have used different protocols of the same tests (Stroop and Go/No-Go) to assess cognitive function, particularly attention and inhibitory control, making it difficult to compare results. Authors have also tested the effect of a single resistance training session using different training regimes (e.g., two sets of one or six resistance exercises and lasting 17 and 45 min) on cognitive function in male and mixed-gender samples. No study has investigated the effect of 30 min of different resistance exercise intensities, with more sets and different types of exercises, on cognitive function and psychological states in females. The fact that females remain underrepresented in scientific studies may be due to the misguided assumptions, such as “female physiology was too complicated”, “men’s and women’s bodies were essentially the same” and “the lower number of females who train and compete professionally makes studies not worth conducting” [22]. This issue is slowly being resolved by the increase in scientific investigations on females, showing that females have a specific and unique training/exercise adaptation/response [22]. For example, a previous study reported that resistance exercise induced significantly higher brain-derived neurotrophic factor (BDNF) response in older males than female counterparts [23].

Attention is an important component in many domains and activities, such as education and sport, and people often have to concentrate on important activities after participating in acute exercise (e.g., students who have academic classes after physical education; athletes who need a high level of attention after such exercise to make better decisions). In sport situations among others, competing conclusions have been reported in the literature regarding the effect of acute exercise intensity on attention [21]. Therefore, it is crucial to determine if and when resistance exercise intensity impacts attention. Additionally of interest is what the best resistance exercise intensity is to improve cognitive performance and consequently improve decision making, which in turn may have beneficial effects for “in the field” performance. For example, if a specific exercise of a specific intensity enhances attention, we can advise students or athletes to practice this exercise before engaging in an activity that requires high levels of attention. Due to the limitations of previous research, the purpose of this study was to examine the acute effect of high- and moderate-intensity strength exercise on attention, using the d2 test of attention, and mood states in female physical education students, including rating of perceived exertion and mood.

## 2. Materials and Methods

### 2.1. Participants

A total of 46 female physical education students (M_age_ = 20.02 ± 1.05 years, M_Body Mass Index_ = 21.07) volunteered to participate in this study after being informed of the nature of the experiment. Participants were team sport athletes (soccer, handball, and volleyball) without having prior experience in resistance training. They were randomly assigned to one of three groups: a moderate-intensity strength exercise group (MISEG: *n* = 15, M_age_ = 20.12 ± 1.10 years, M_Body Mass Index_ = 20.97), a high-intensity strength exercise group (HISEG: *n* = 16, M_age_ = 19.50 ± 1.15 years, M_Body Mass Index_ = 22.16), and a control group (CG: *n* = 15, M_age_ = 19.82 ± 1.04 years, M_Body Mass Index_ = 21.03), according to their one-repetition maximum (1RM). We used an independent groups design in accordance with previous studies [18,19].

Participants who presented the following condition(s) were excluded: (a) co-morbidities and mental health problems. Those presenting the following condition(s) were included: (a) being physically active (doing a regular or structured exercise program in or outside school(s) except for daily walking) from 48 h before the experimental conditions (as will be detailed below); and (b) refraining from coffee and any strenuous exercise 48 h before the days of any trial. The study was conducted according to the guidelines of the Declaration of Helsinki, and was approved by the Ethics Committee of Taif University, Saudi Arabia and by the UNESCO Chair “Health Anthropology Biosphere and Healing Systems,” University of Genoa, Genoa, Italy (project code EXERCOGN_023020). All participants gave their consent to participate in this study by signing a consent form.

### 2.2. Protocol

Participants visited the laboratory on two separate occasions. Before the experiment, participants were invited to a first session in which they were informed about differences between moderate and high-intensity exercise and the study’s details: the training protocol consisted of the 3 sets of 5 exercises targeting all the major muscle groups of the body. Then, the psychological measures used in the study were presented and explained to them. Anthropometric data were also collected during the first visit. In addition, a 1RM was estimated indirectly for each exercise group [24], according to the guidelines of the National Strength and Conditioning Association [25].

During the second session, all participants completed a cognitive performance test (d2 test) and the psychological questionnaires [Rating of Perceived Exertion (RPE), mood] before completing one of the three conditions (moderate-intensity strength exercise session, high-intensity strength exercise session, or the control session). They performed a 10 min warm-up and cool-down (e.g., running, jumping rope, arm circles, hip rotations, squats, push-up, etc.) before and after each strength exercise session.

For the moderate-intensity strength exercise, subjects performed 3 sets of 8–10 repetitions with a training intensity of 55% 1RM. For the high-intensity strength exercise, participants performed 3 sets of 6 repetitions with a training of intensity 80% 1RM [26,27]. Participants were granted 120 s of rest between sets and exercises. The exercises performed during both sessions were: flat barbell press, wide grip lat pull down, seated cable row, machine leg press, and machine leg extension. In the control group, participants read information about the health benefits of strength exercise. Each session for the three conditions/groups (MISEG, HISEG and CG) lasted approximately 30 min. Immediately after each session, all participants completed the RPE and the Brunel Mood Scale (BRUMS) questionnaires again after performing the d2 test.

### 2.3. Attention Assessment

The d2 test was used to determine the level of concentrated visual attention of participants [28]. It consists of 14 rows with 47 characters per line. These characters are the letters “d” or “P”, with a total of one to four dashes above and below each letter. Participants were asked to scan each line and cross out only the characters containing the letter d with two dashes during 20 s.

After completion of the d2 test, two variables were calculated: concentration performance (CP) and total number of errors made by the participants (E). CP is calculated as the number of correctly marked d2-symbols minus the number of incorrectly marked symbols (symbols that are not d2-symbols). The total number of E is assessed as the number of errors subjects made by failing to correctly identify a d2-symbol plus the number of errors made by incorrectly marking symbols that are not d2-symbols.

### 2.4. Rating of Perceived Exertion (RPE)

Before and after each session, the RPE scale was used to estimate the participants’ perceived effort. It ranged between 0 “no perceived effort” (i.e., rest) and 10 corresponded to “maximal perceived effort” (i.e., the most stressful exercise ever performed) [29]. Of note, the reliability of RPE scale was excellent [intraclass correlation coefficient (ICC) = 0.83].

### 2.5. Mood

Mood state was assessed using the BRUMS developed by Terry et al. [30] and involved asking the question “How do you feel right now?” after each session. This questionnaire contains 24 items divided into six respective subscales: anger, confusion, depression, fatigue, tension, and vigour. The items are answered on a 5-point Likert scale (0 = not at all, 1 = a little, 2 = moderately, 3 = quite a bit, and 4 = extremely), and each subscale, with four relevant items, can achieve a raw score in the range from 0 to 16. The ICC reliability of BRUMS subscales ranged between 0.72 and 0.85.

### 2.6. Statistical Analysis

The sample size needed was calculated by performing an a priori power analysis by means of the open-source software G*Power (version 3.1.9.7, Kiel, Germany), setting the alpha error probability at 0.05, the power (1—beta error probability) at 0.80, with three groups and two time-points. To detect a medium-large effect size, assuming a medium correlation between the repeated measures, the minimum sample size needed to consist of 45 subjects.

Descriptive statistical analysis was carried out by computing the means and standard deviations for each of the variables under study. Normality of data distribution was verified by applying the Shapiro–Wilk test, which was preferred over other normality tests, given the sample size employed in the present investigation. Analysis of variance (ANOVA), or its non-parametric version depending on the normality of data, was carried out to capture differences between the BRUMS measures among the groups.

A repeated-measures ANOVA (rmANOVA) with a grouping factor was utilized to quantify the effects of moderate versus high-intensity exercise on attention-related measures (CP and E) and on RPE scores. Besides verifying the normality of distribution, the homogeneity of covariance matrices, as well as the independence and the sphericity assumptions, were checked. More in detail, Mauchly’s test was run to check for sphericity. In case of a violation of this assumption, the Greenhouse–Geisser correction was applied to account for the degrees of freedom of the interaction effect between the various time points and the groups. If the figure was less than 0.75, the Huynh–Feldt correction was utilized. Post-hoc tests were conducted utilizing Bonferroni correction. The effect size (ES) was categorized as small (0.01 < η^2^ < 0.06), medium (0.06 < η^2^ < 0.14) or large (η^2^ > 0.14) [15]. All statistical analyses were conducted utilizing the commercial software “Statistical Package for Social Sciences” (SPSS version 24.0, IBM, Armonk, NY, USA). Results with *p*-values less than or equal to 0.05 were considered statistically significant.

## 3. Results

Descriptive values are reported in Table 1 for CP, E and RPE, whereas the scores obtained from the BRUMS questionnaire are shown in Table 2 of the overall sample. Concerning attention and RPE, CP values (in terms of pairwise comparisons) differed between before and after (F = 4.73, *p* = 0.035), but did not differ among the groups (F = 1.07, *p* = 0.351, ES = 0.05, small). The group x time interaction effect was not significant (F = 2.61, *p* = 0.086). E values did not differ in terms of group (F = 0.87, *p* = 0.427, ES = 0.04, small), or time (F = 2.47, *p* = 0.124). The group x time interaction effect was also not significant (F = 1.28, *p* = 0.290). RPE values, however, were different in terms of group (F = 8.69, *p* = 0.001, ES = 0.29, large), being higher for the HISEG than the CG (*p* < 0.001) and MISEG (*p* = 0.008) (Table 3). Moreover, the effects of time (F = 283.49, *p* < 0.001) and time x group interaction (F = 9.88, *p* < 0.001) were also significant.

Regarding the BRUMS scale, fatigue (*p* < 0.001) was significantly different among groups, being higher in HISEG with respect to controls and MISEG (*p* < 0.05). Similarly, confusion differed among groups (*p* = 0.005) and was significantly higher in HISEG compared to MISEG (*p* < 0.05). Vigour varied among groups (*p* < 0.001) with MISEG reporting higher scores with respect to controls and HISEG (*p* < 0.05). Noteworthy, anger was another sub-scale differing among groups (*p* = 0.009), being higher in CG with respect to MISEG (*p* < 0.05). All remaining sub-scales did not significantly vary among groups (Table 4).

## 4. Discussion

The data showed that for moderate-intensity strength exercise, attention, in terms of CP, increased significantly. However, RPE and the fatigue and confusion subscales were higher in the HISEG than the MISEG and the CG. The vigour subscale was also higher in MISEG than the other groups.

Many studies have reported that acute resistance exercise improves cognitive function [21,31]. However, many cognitive subdimensions (i.e., attention, working memory, cognitive flexibility, inhibitory control) were used to assess cognitive performance after 20–45 min of resistance exercise [21]. Some studies have found that executive function (e.g., the Stroop test and the Tower of London task), in terms of reaction time and inhibitory control, was higher after resistance exercise when compared with pre-exercise and no-exercise interventions [7,32,33,34]. Another study reported that moderate-intensity resistance and aerobic exercise have a positive impact on simple reaction time, working memory, response precision, basic information processing and the inhibitory aspect of executive function [35,36]. These findings are in accordance, in part, with our results, which showed that only moderate-intensity strength exercise increased cognitive function, particularly CP. Nevertheless, the effect size and benefits of resistance exercise varied depending on the exercise intensity [18,37]. Chang and Etnier [18] reported a linear relationship between resistance exercise intensity and information processing speed. They showed that 30 min of moderate-intensity exercise has the most beneficial effect for executive function, as assessed using the Stroop test, which assessed reaction times and the accuracy by verbally responding to each word that was printed with the same or different ink colour, when compared with light- and high-intensity resistance exercises. Similarly, Chang et al. [37] showed that reaction time and cognitive performance as assessed by the Stroop test (neutral task) were higher following 15 min of moderate-intensity exercise than high-intensity exercise. In contrast, the present study showed that there is no significant difference between moderate- and high-intensity resistance exercise for the d2 test performance. These findings are not consistent with previous literature regarding the inverted-U relationship between exercise intensity and cognitive function. This contradiction may be due to the participant’s cardiorespiratory fitness [38]. Of note, those with a higher level of physical fitness recover rapidly from high-intensity exercise than those with a lower level [39].

Based on the previous studies, it appears that resistance/strength exercise of 50–75% of 1RM is the most effective exercise intensity to improve cognitive function. From a physiological point of view, exercise of moderate intensity increases blood flow to the cerebrum and elevates haemoglobin levels, which in turn improves cognitive function [39]. Other potential moderating factors are the higher concentrations of neurotransmitters in the brain [40], and cellular processes like neural generation (neurogenesis) and activation, survival of neurons (neuroprotection), synaptic plasticity, growth, or general physiological arousal [16,21]. Furthermore, moderate-intensity resistance exercise seems to modulate the levels of insulin growth factor 1 (IGF-1) and neurotrophins, such as the BDNF at the level of the striatum, hippocampus and other cortical regions [41,42]. Another important finding from the present study is that high-intensity resistance exercise did not affect cognitive function. This could be explained by high-intensity exercise increasing cerebral blood flow and neurotransmitter release, including BDNF [43,44], which in turn elevates stress levels beyond the anaerobic threshold, which subsequently interferes with cognitive function [40].

From a psychological point of view, it was shown that the vigour subscale was higher immediately after moderate intensity, rather than high-intensity resistance exercise. The elevated vigour scores may lead to increased CP after moderate-intensity exercise. In addition, the present data showed that RPE, fatigue and confusion subscales increased with the exercise intensity. A possible explanation for the increase in RPE responses has previously been proposed as a feed-forward mechanism that links perceived exertion with muscle activity [45] and the peripheral component of the stretch reflex [46]. It has also been previously reported that there is greater force development, which requires an increase in motor unit recruitment and firing frequency, when muscles are submitted to heavy loads [47]. The increase in muscle activity triggers stronger motor cortex signals to the sensory cortex, which may increase the RPE [48]. However, contradictory results have been shown in the literature regarding the relationship between session-RPE and training intensity and total work performed. Some studies have suggested that RPE and session RPE are affected by the total volume of load lifted (TVLL) more than the intensity when multiple sets are performed until volitional exhaustion [49,50]. McGuigan [51] showed that as the total training volume and intensity during resistance exercise increased, the perception of effort of the participants also increased. In contrast to previous findings, Day et al. [52] compared the session RPE response to three different workouts involving five exercises (1 set each) using loads of 50% 1RM (15 repetitions), 70% 1RM (10 repetitions), and 90% 1RM (5 repetitions). They found that the session RPE increased concurrently with the percentage of 1RM lifted.

The present study added new evidence on acute cognitive and mood states responses to 30 min of moderate-intensity resistance exercise in comparison with high-intensity resistance exercise. The findings may provide useful information in the real world. For example, physical educators or exercise instructors may (a) implement moderate-intensity resistance exercise to improve attention and (b) avoid practising/including high-intensity resistance exercise before any activity that requires a high level of attention and vigour. If these suggestions are not possible, some strategies might be employed to reduce the feelings of fatigue and accelerate cognitive recovery after high-intensity resistance exercise.

This study has several limitations. First, we recruited female adults and thus findings may not be applicable to other populations (i.e., children, aged people, males, patients or individuals with medical conditions). Second, we classified the intensity of resistance exercise based on an estimation of 1RM rather than a direct measurement, which may influence the results. However, the estimation method of 1RM is of low risk, safe and adequate for our study population. Third, given that previous and present studies have mostly focused on the impact of acute resistance exercise on cognitive function using laboratory tests (such as the d2, Stroop and Go/No-Go tests), future research may investigate the effect of acute resistance exercise on “in-field” performance. Finally, we did not assess the energy expenditure of participants to compare our data with previous studies’ results.

In conclusion, the present study showed that only acute moderate-intensity resistance exercise (session lasting approximately 30 min of 55% 1RM) has an impact on attention, particularly concentration performance and, as such, can be an appropriate method to improve attention in female participants. The possible mechanisms underlying the relationship between resistance exercise intensity and enhanced attention could be changes in RPE and in mood state, in particular fatigue, vigour and confusion. However, further research is warranted to replicate our findings in a statistically robust way and both short- and long-term effects of acute intensity resistance exercise should be studied. Additionally, different populations (including non-athletes) should be explored.

## Figures and Tables

**Table 1:** Descriptive statistics of the variables under study for the overall sample.

Variable		Value	
		Mean	SD
CP	CP after	74.72	15.21
	CP before	70.44	12.75
E	E after	19.20	13.83
	E before	22.35	11.69
RPE	RPE after	3.67	1.56
	RPE before	0.46	0.75

CP: concentration performance, E: errors, RPE: Rating of Perceived Exertion, SD: standard deviation.

**Table 2:** Descriptive statistics of the scores of the Brunel Mood Scale (BRUMS) questionnaire for the overall sample.

Variable	Item/Sub-Scale	Value	
		Mean	SD
Anger	Anger 1	1.11	0.43
	Anger 2	1.26	0.57
	Anger 3	1.52	1.17
	Anger 4	1.07	0.25
	Total sub-scale	4.96	1.43
Confusion	Confusion 1	1.46	0.86
	Confusion 2	1.30	0.70
	Confusion 3	1.52	0.84
	Confusion 4	1.28	0.58
	Confusion sub-scale	5.57	2.43
Depression	Depression 1	1.22	0.66
	Depression 2	1.11	0.48
	Depression 3	1.02	0.15
	Depression 4	1.04	0.21
	Depression sub-scale	4.39	1.02
Fatigue	Fatigue 1	1.41	0.65
	Fatigue 2	1.29	0.46
	Fatigue 3	1.52	0.62
	Fatigue 4	1.46	0.62
	Fatigue sub-scale	5.65	1.78
Tension	Tension 1	1.17	0.44
	Tension 2	1.09	0.35
	Tension 3	1.07	0.33
	Tension 4	1.30	0.59
	Tension sub-scale	4.61	1.13
Vigour	Vigour 1	2.65	1.18
	Vigour 2	2.72	1.13
	Vigour 3	2.91	1.07
	Vigour 4	2.96	1.10
	Vigour sub-scale	11.24	3.88

SD: standard deviation.

**Table 3:** Values of concentration performance (CP), errors (E), and rating of perceived exertion (RPE) broken down according to the group.

Variable		MISEG	HISEG	CG	Statistical Significance of the Group Effect	Statistical Significance of the Time Effect	Statistical Significance of the Group × Time Interaction Effect	Effect Size
CP	Before	70.87 ± 10.06	68.44 ± 13.51	72.13 ± 14.76	F = 1.07 (*p* = 0.351)	F = 4.73 (*p* = 0.035)	F = 2.61 (*p* = 0.086)	0.05
	After	81.40 ± 18.31 *	71.19 ± 9.92	71.80 ± 15.23				
E	Before	21.87± 11.92	22.94 ± 13.45	22.20± 10.12	F = 0.87 (*p* = 0.427)	F = 2.47 (*p* = 0.124)	F = 1.28 (*p* = 0.290)	0.04
	After	14.53 ± 18.26	23.63 ± 12.99	19.13 ± 7.43				
RPE	Before	0.27 ± 0.70	0.63 ± 0.81	0.47 ± 0.74	F = 8.69 (*p* = 0.001)	F = 283.49 (*p* < 0.001)	F = 9.88 (*p* < 0.001)	0.29
	After	3.40 ± 1.30 ***,£	4.88 ± 1.54 ***,#	2.67 ± 0.90 ***				

*** Significant difference compared with before exercise at *p* < 0.001, * Significant difference compared with before exercise at *p* < 0.05, # Significant difference compared with CG at *p* < 0.001, £ Significant difference compared with HISEG at *p* < 0.05, CG: control group, CP: concentration performance, E: errors, HISEG: high-intensity strength exercise group, MISEG: moderate-intensity strength exercise group, RPE: Rating of Perceived Exertion.

**Table 4:** Scales of the Brunel Mood Scale (BRUMS) scale broken down according to the group.

Variable		MISEG	HISEG	CG	Statistical Significance
Anger	Anger 1	1.07 ± 0.26	1.00 ± 0.00	1.27 ± 0.70	NS
	Anger 2	1.13 ± 0.35	1.19 ± 0.40	1.47 ± 0.83	NS
	Anger 3	1.00 ± 0.00	1.56 ± 1.09	2.00 ± 1.60	NS
	Anger 4	1.07 ± 0.26	1.06 ± 0.25	1.07 ± 0.26	NS
	Anger sub-scale	4.27 ± 0.46	4.81 ± 1.22	5.80 ± 1.86	*p* = 0.009
Confusion	Confusion 1	1.07 ± 0.26	1.88 ± 1.20	1.40 ± 0.63	*p* = 0.028
	Confusion 2	1.07 ± 0.26	1.63 ± 0.96	1.20 ± 0.56	NS
	Confusion 3	1.07 ± 0.26	1.81 ± 1.11	1.67 ± 0.72	*p* = 0.029
	Confusion 4	1.00 ± 0.00	1.63 ± 0.81	1.20 ± 0.41	*p* = 0.007
	Confusion sub-scale	4.20 ± 0.56	6.94 ± 3.38	5.47 ± 1.51	*p* = 0.005
Depression	Depression 1	1.13 ± 0.35	1.00 ± 0.00	1.53 ± 1.06	NS
	Depression 2	1.00 ± 0.00	1.19 ± 0.75	1.13 ± 0.35	NS
	Depression 3	1.00 ± 0.00	1.00 ± 0.00	1.07 ± 0.26	NS
	Depression 4	1.00 ± 0.00	1.00 ± 0.00	1.13 ± 0.35	NS
	Depression sub-scale	4.13 ± 0.35	4.19 ± 0.75	4.87 ± 1.51	NS
Fatigue	Fatigue 1	1.20 ± 0.41	1.88 ± 0.81	1.13 ± 0.35	*p* = 0.001
	Fatigue 2	1.21 ± 0.43	1.50 ± 0.52	1.13 ± 0.35	NS
	Fatigue 3	1.40 ± 0.51	1.94 ± 0.68	1.20 ± 0.41	*p* = 0.002
	Fatigue 4	1.27 ± 0.46	1.75 ± 0.68	1.33 ± 0.62	NS
	Fatigue sub-scale	5.00 ± 1.36	7.06 ± 1.69	4.80 ± 1.32	*p* < 0.001
Tension	Tension 1	1.20 ± 0.41	1.06 ± 0.25	1.27 ± 0.59	NS
	Tension 2	1.07 ± 0.26	1.00 ± 0.00	1.20 ± 0.56	NS
	Tension 3	1.13 ± 0.52	1.00 ± 0.00	1.07 ± 0.27	NS
	Tension 4	1.27 ± 0.59	1.44 ± 0.73	1.20 ± 0.41	NS
	Tension sub-scale	4.67 ± 1.18	4.50 ± 0.82	4.67 ± 1.40	NS
Vigour	Vigour 1	3.60 ± 1.06	2.56 ± 1.03	1.80 ± 0.68	*p* < 0.001
	Vigour 2	3.60 ± 1.18	2.44 ± 1.03	2.13 ± 0.52	*p* < 0.001
	Vigour 3	3.60 ± 1.18	2.88 ± 1.02	2.27 ± 0.46	*p* = 0.002
	Vigour 4	3.73 ± 0.96	2.88 ± 1.20	2.27 ± 0.46	*p* < 0.001
	Vigour sub-scale	14.53 ± 4.05	10.75 ± 3.09	8.47 ± 1.25	*p* < 0.001

CG: control group, HISEG: high-intensity strength exercise group, MISEG: moderate-intensity strength exercise group, NS: not significant.

## Data Availability

The data are not publicly available due to privacy or ethical reasons.
